# Formative Qualitative Research: Design Considerations for a Self-Directed Lifestyle Intervention for Type-2 Diabetes Patients Using Human-Centered Design Principles in Benin

**DOI:** 10.3390/ijerph191811552

**Published:** 2022-09-14

**Authors:** Halimatou Alaofè, Abidemi Okechukwu, Sarah Yeo, Priscilla Magrath, Waliou Amoussa Hounkpatin, John Ehiri, Cecilia Rosales

**Affiliations:** 1 Department of Health Promotion Sciences, Mel and Enid Zuckerman College of Public Health, The University of Arizona, Tucson, AZ 85724, USA; 2 School of Nutrition and Food Science and Technology, Faculty of Agricultural Sciences, University of Abomey-Calavi, Abomey-Calavi 01 BP 526, Benin; 3 Division of Public Health Practice & Translational Research, University of Arizona, Phoenix Plaza Building, 550 E. Van Buren Street, Phoenix, AZ 85006, USA

**Keywords:** type 2 diabetes, human-centered design, culturally tailored interventions, Meta-Salud diabetes, Benin

## Abstract

Given the burgeoning prevalence of Type-2 Diabetes (T2D) in Benin and other sub-Saharan countries, tailored diabetes self-management interventions are urgently needed. Human-centered designs can be useful for identifying beneficiaries’ needs while keeping in mind feasibility and viability in a given context. Therefore, this study examined the acceptability and community perceptions of a self-directed lifestyle program for T2D patients in Cotonou, southern Benin. Data were collected using focus group discussions (FDGs) with T2D patients (n = 3; 32 participants), academic partners (n = 2; 16 participants), and community partners (n = 2; 12 participants). All FDG sessions were audio-recorded, transcribed from French into English verbatim, and analyzed using MAXQDA 2020. Most participants found the program to be useful and feasible. However, they preferred pictorial brochures as training materials and suggested community health workers as facilitators, assisted by clinicians or dietitians. They recommended community-based delivery mechanisms and mobile applications like WhatsApp to enhance patient adherence. Participants’ characteristics, tangible health benefits, incentives, and simple curriculums were cited as critical to program feasibility, effectiveness, and acceptability. This study provides a deeper understanding of potential diabetes self-management participants’ needs and concerns. Moreover, it highlights the need to consider key stakeholders’ needs and voices for effective intervention.

## 1. Introduction

Globally, the prevalence and impact of Type 2 diabetes (T2D) have increased dramatically [1], and the Republic of Benin is no exception. In the last decade, diabetes prevalence in Benin quadrupled from 3% to 12.4%, reaching 21.6% in some areas [2]. The country faces unique challenges in fighting the disease, including inadequate funding, a lack of research and guidelines tailored to the population, and a shortage of medications [3,4]. Few T2D patients followed dietary guidelines (20%), exercised regularly (<40%), and adhered to prescribed treatments (10%) [5,6]. As a result, diabetes-related disabilities increased by 55.8% between 2007 and 2017. The diabetes-standardized death rate for women rose to 618 and for men to 430 [7,8], indicating the need for contextualized strategies to manage diabetes.

Advances in T2D management indicate that medical treatments combined with sustained lifestyle changes such as regular exercise and a healthy diet significantly improve patients’ glycemic control and overall health outcomes [9,10]. There is evidence that self-directed interventions that empower people with diabetes can effectively control biochemical and physical parameters. In addition, these combined interventions can be scaled up across the population to improve health outcomes and health system performance [11,12]. However, long-term management of T2D can quickly become costly and tedious for patients, often forcing them to share the burden with family members and the community.

The fatigue from long-term management of T2D sometimes deters patients’ adherence to medical treatments and lifestyle recommendations leading to worse glycemic and health outcomes. The negative impacts of non-adherence to T2D therapies and lifestyle recommendations are particularly severe among populations living in resource-constraint settings, where social safety nets are almost non-existent, and/or where remedial clinical treatments are not accessible to most of the population [9]. The considerable burden of poorly managed T2D in low-resource contexts establishes the imperative to design and implement disease management strategies responsive to people’s needs and well adapted to constraints within their contexts. Human-Centered Design (HCD) technique provides a qualitative approach to designing complex interventions that offer relevant solutions tailored to the needs and contexts of T2D patients.

The HCD approach combines empathy and co-creation to identify the needs of beneficiaries while considering viability and feasibility in the design of an intervention [13,14]. The HCD technique considers three stages of the design process: Inspiration, Ideation, and Implementation [13,15,16]. Inspiration is usually the first step in the design process that aims to understand the preferences of the intended users or beneficiaries. The design team identifies a design challenge and employs various qualitative methods to gather information from potential beneficiaries or the community. Next, the Ideation stage uses the insight gained from Inspiration to design intervention prototypes, and the Implementation stage involves the process of implementing the new or adapted intervention design.

The HCD approach has been successfully applied to diabetes education in global health [17,18,19,20,21]. Many studies exploring diabetes management education have identified three critical issues to consider when designing lifestyle interventions for people with diabetes. These include the need: (1) for people with diabetes to share their lifestyle changes and experiences with their family and community [17], (2) for interventions to consider a broader range of personas and lifestyles in designing interventions [19], and (3) to co-create diabetes management interventions with inputs from beneficiaries and their healthcare providers as a team [20,21,22]. The objective of this qualitative study was thus to determine the needs and preferences of T2D patients, community health workers, and healthcare providers to inform the design of a self-directed T2D management curriculum using HCD techniques in Cotonou, Republic of Benin.

## 2. Materials and Methods

Study setting: The study was carried out in Cotonou, the largest city in the Republic of Benin. Cotonou has a population of 1.2 million, and French is the official language [23]. Benin is ranked 158th out of 189 countries on the 2020 Human Development Index [24]. Approximately 49.5% of the population earned less than 2 USD a day; 72% completed primary school education, while only 1% had tertiary-level education. The literacy rate is low; 80% of rural and 47% of urban residents cannot read French [23]. Additionally, in the 2015 Benin, WHO STEPwise approach to non-communicable disease surveillance (STEPS), the prevalence of diabetes increased from 4.4 to 19% in the last decade [2].

Study design: This qualitative study was conducted in July and August 2019 using seven focus group discussions (FGDs) to learn their perspectives on the framework, approach, acceptability, and recommendations for a diabetes self-management education program. Three FGDs were conducted with patients with T2D, two with community partners, and two with academic partners. In addition, a literature review of previous evidence-based self-directed interventions in Benin-like settings led to the current study’s use of the Meta Salud Diabetes (MSD) program [25,26].

Meta Salud Diabetes (MSD) Curriculum: Although there are successful and popular self-directed diabetes interventions such as DESMOND, most were developed and delivered in developed countries that are very different from rapidly developing countries regarding their health systems, culture, traditions, and lifestyle behaviors related to nutrition and diet. Most of these programs were also undertaken in countries with a robust enabling environment of policy and other supports for preventing and controlling chronic non-communicable diseases. The MSD is a chronic disease prevention program that targets uninsured people living with diabetes in Sonora, Mexico. MSD is informed by two behavioral change theories, the Trans Theoretical Model of Behavior Change and the Social Cognitive Theory. According to these two theories, behavior modification is a multi-stage process that involves people moving through stages of readiness for change and engaging in reciprocal relationships with their environment, behavior, and cognition [27,28]. The 13-week program consisted of modules held in two-hour sessions that stressed nutrition and physical activity and addressed social support, health behavior, and emotional well-being [25]. MSD program has been evaluated across a culturally diverse and underserved population in Mexico in a cluster-randomized controlled trial that shows the intervention significantly reduces the symptoms of diabetes among patients in the treatment arm after 3 and 12 months of intervention [26]. We argue that the MSD curriculum is feasible and practical to adapt in Benin, given the country’s low access to health education for people with diabetes [6,29]. Moreover, the community setting in Benin provides a unique opportunity to leverage family and community support for improving self-directed diabetes management.

Sampling and procedure: Purposeful sampling was performed until data saturation. We selected community partners for FGDs based on their direct involvement in disease education and management. FGDs with community partners involved ten community health workers and two traditional healers. In addition, FGDs were conducted with 16 healthcare providers to learn their perspectives on the program based on their client experience. They represented various professions, including four endocrinologists, four general practitioners, four pharmacists, and four nutritionists. As for T2D patients, 32 individuals participated in FGDs. Patients were all over 40 years of age and grouped by educational status to facilitate open dialog within groups: one FGD with primary level education and two with secondary and above education.

Each of the seven FGs lasted 1.5-2 h and followed a semi-structured focus group protocol. Participants answered the same questions, allowing for data comparison across participant groups [30]. All three groups were invited to a community center and given an orientation on the MSD program, designed to help individuals with diabetes maintain their blood glucose levels at an optimal level. After the presentation, participants had the opportunity to discuss their perceptions, preferences, and recommendations for adapting the MSD module and intervention to the Benin context. The lead author (HA) facilitated all data collection with two research assistants (RAs), which participated in a two-day training on consent procedures, FGD methods, and research ethics.

Ethical considerations: This study was approved by the National Ethics Committee for Health Research (CNRES) of Benin. Ethical clearance was also obtained from the institutional review boards (IRB) charged with the Human Subjects Protection Program (HSPP) of the University of Arizona (IRB 1508040144). The study researchers informed participants about the aims and procedures of the study and written informed consent was obtained from all participants before enrollment.

Data analysis: FGDs were audio-recorded, transcribed, and translated from French to English by two research team members. Translated transcripts were reviewed, and a codebook was developed in response to the data review. The transcripts were coded using VERBI Software, MAXQDA 2020, software 2019 (Berlin, Germany), maxqda.com [31], and analyzed using thematic analysis methodology [32]. The analysis included both deductive, informed by the research questions and discussion guides, and inductive themes, identified by reviewing transcripts. The themes were divided into subthemes, reviewed, modified, and approved by all the authors. Lastly, code summaries were developed by reviewing data coded for each theme and separated by components.

## 3. Results

In total, seven FGDs were held with 60 participants. Participants included T2D patients aged 40 and over (n = 3 FGDs; 32 participants), community partners (n = 2 FGDs; 12 participants), and academic partners (n = 2 FGDs; 16 participants). Most participants (80%) in all three groups were married. About 62% of the academic group participants were male, while 58% of the community partners were female. Finally, most academic partners (63%) were government employees, whereas the patients (44%) and community partners (67%) were largely non-government employees.

**Figure 1:**
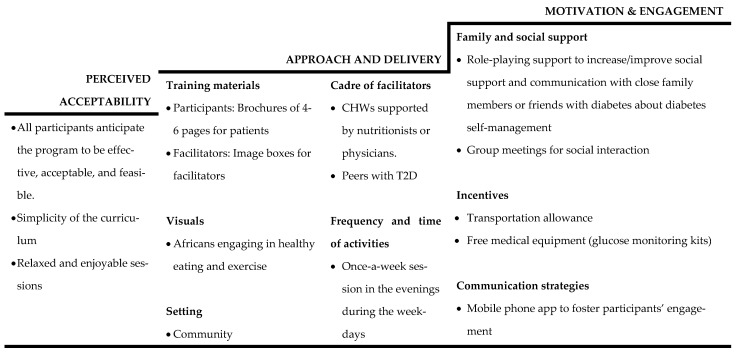
Summary of findings.

The key themes identified are approach and delivery, motivation and engagement, and perceived acceptability. The theme of approach and delivery includes materials, facilitators, venues, frequency and time, and communication strategies. The motivation and engagement theme is related to participation and incentives that could potentially motivate and engage participants (Figure 1).

### 3.1. Approach and Delivery

MSD was designed to be an instructor-led module delivered over thirteen weeks. Approach and delivery consisted of procedures and resources used to design and develop learning experiences or behavior change [33].

Subtheme 1: Module brochure and training materials

Participants from all three groups advocated for color and image-based modules. A majority of participants mentioned using image boxes to facilitate weekly modules. Furthermore, the patient focus groups emphasized the need for cultural and language nuances when adapting the program modules. Program participants would probably try physical activities if the models in the modules looked like people of Benin. Participants stressed the importance of modules in common local languages such as Yoruba and Fon instead of French.

*“I agree with an information brochure of three or four pages that can help us revise everything that has happened.”*—Patient focus group

*“In the village, if they see someone who looks like them doing the exercises [in the brochure], they automatically think they can do them.”*—Community partner focus group

Subtheme 2: Facilitator characteristics

Participants in all three groups identified the Community Health Workers (CHW) as the most feasible and vital facilitator for the modules. Furthermore, participants described the need for support from dietitians/nutritionists, physicians, and other specialists, such as physical fitness specialists, since CHWs may not have all the knowledge necessary to answer specific technical questions from program participants. More importantly, some participants suggested that CHW with diabetes should facilitate the module groups and that these professionals should be connected to local healthcare providers.

*“CHWs [should be facilitators] since they are a link between the doctor and the population. They will have the doctor’s information and know-how to interact with the population. They also know what is going on.”*—Community Partner Focus Group

Subtheme 3: Location of training and activities

Across the three participant groups, most strongly disapproved of using health facilities and hospitals as locations for program delivery when asked explicitly about the best venue to ensure accessibility and participation. Participants believed that hospitals and health centers were associated with illness and death, making them less suitable for this program. Participants suggested various locations for implementing the program, such as Catholic churches, small city halls, and town halls. Participants throughout the groups echoed the importance of the training location’s proximity to the community.

*“The hospital in the collective mind is a dying place [a place where people die]—so you will not see many people.”*—Community Partner Focus Group

Subtheme 4: Proposed frequency and time of program activities

Most participants in all three groups agreed that a once-a-week session would be most feasible and desirable for participants and trainers. In all three groups, participants reported their preference for evening classes during the weekdays since they were busy or working during the day.

*“I think once a week for two hours is okay if it is a participatory session. If you invite people for an hour each day, twice a week, it already takes time [to attend one meeting]”*—Patient Focus Group

Subtheme 5: Communication strategies

Participants in all three groups stressed the need for a forum to communicate, voice concerns, and share ideas and suggested using mobile social media platforms such as WhatsApp.

*“[We need to] have a forum on WhatsApp where we can communicate, and we can advise each other, and if you have any concerns, we can share this.”*—Patient Focus Group

### 3.2. Motivation and Engagement

Diabetes self-care behavior models define motivation as positive personal beliefs, attitudes, and social support toward a behavioral outcome [34]. Motivation with prerequisite skills is key to sustaining positive behavior or engagement. Engagement is the act of using an intervention or the tendency to interact with it, including factors that influence that behavior [35]. A qualitative analysis of engagement may help explain how users respond to an intervention. Participants suggested that tangible health improvement and benefits would motivate and encourage patients, stressing the importance of an awareness session before the program.

*“Results [including] health improvements that we [participants] will observe will motivate and encourage us to attend the meetings until the end.”*—Patient Focus Group

Subtheme 1: Participation

Many participants indicated their preference for participation in the proposed intervention by members of their immediate family or friends who also have diabetes. Comments across participant groups converged on the idea that family and friends with diabetes would be more empathic and supportive. A participant from the academic partner noted that accompanying a family member increases adherence to clinical advice and medications.

*“I prefer participants to be people who have diabetes or my family.”*—Patient Focus Group.

Subtheme 2: Incentives

Patient focus groups had diverging views about their motivations and incentives. Some participants argued that they would not need inspiration as they already prioritized health, among other things. However, participants from the community partner and academic partner groups hinted those incentives such as transportation allowance or glucose monitoring kits could motivate the patients given the financial constraints in the context.

*“I think that incentives such as free medical equipment to use [glucometers and blood tests] can also motivate them. Transportation costs too.”*—Academic Partner Focus Group

### 3.3. Perceived Acceptability

Acceptability refers to the desire for appropriate and effective interventions to address health problems [36]. Perceived acceptability will improve our understanding of what beneficiaries expect for a successful program or intervention. Participants in the patient FGDs agreed that the adapted program would generally be agreeable to beneficiaries because such a curriculum will be innovative within the Cotonou context. However, participants from the patient FGD expressed concerns about sustainability. Participants mentioned that an easy-to-implement curriculum would improve participants acceptance of the MSD program. In addition, participants in the community partners’ FGD noted that engaging participants in a relaxed and enjoyable mode would improve participants’ receipt and use of the adapted MSD curriculum.

*“No problem, if the questions are understandable and there are answers to be ticked, and these are simple reports to present”*—Patient Focus Group

## 4. Discussion

This study led to a more nuanced understanding of the needs of people living with diabetes in Benin, especially in Cotonou, and provided knowledge that will guide the adaptation and implementation of a self-directed T2D management curriculum in the country. Using HCD approaches, we examined the feasibility, acceptability, and curriculum adaptation transformations needed for the MSD program to address the increased burden of diabetes in the country. Among the factors, they noted that training materials, facilitator characteristics, approach to program delivery, and communication strategies needed to be tweaked to improve their feasibility and acceptability. Furthermore, study participants agreed that the program participants’ incentives, the curriculum’s simplicity, and the process of engaging program participants would play a critical role in the MSD program’s acceptability, feasibility, and effectiveness.

The MSD curriculum was facilitated by hospital-based health professionals such as physicians and nurses in Mexico. However, this is not practical in Cotonou due to higher costs and a shortage of health professionals [3]. The participants in this study indicated that CHWs would be better suited as facilitators, supported by clinicians and dietitians. The finding supports evidence from several studies indicating how task-shifting enhances treatment access and reduces costs [37]. Alaofe et al. [38] also found that CHWs in low- and middle-income countries can improve knowledge, health behavior, and health outcomes related to preventing and managing T2D. Respondents suggested locating the program near the target population and including facilitators who have diabetes. In a review of studies that included family members in self-care interventions, Baig et al. [39] reported significant improvements in their self-efficacy, perceived social support, diabetes knowledge, and self-care with diabetes when family members are involved in care. Similarly, peer education is a popular health promotion and disease prevention strategy [40].

Furthermore, curriculum content should be tailored to the literacy level of facilitators and participants, considering participants’ preferences for the local language. It is essential given that many people with poor diabetes management report low levels of education. Specifically, in Benin, only 1% of the population had tertiary education, and 47% of urban residents could not read French [23]. Low levels of education may be associated with participants’ preferences toward image-based modules and facilitation. Wollny et al. [41] found that written information provided by general practitioners to patients with diabetes had little effect on metabolic control. Moreover, studies evaluating picture-based health education tools found them effective in low health literacy settings, affirming that pictographic health education is adequate irrespective of respondents’ race or ethnicity [42,43]. Benjamin et al. even pointed out that health literacy could play an essential role in reducing disparities [44].

Aside from the barriers, some facilitators may provide the support needed to implement the intervention in the study area. Participants preferred group-based sessions, which offer opportunities to tap existing social networks and community-based health infrastructure. This finding provides a closer look at the challenges associated with diabetes care in Benin. Studies in Africa reveal several challenges in accessing care and adhering to treatment due to patients’ lack of knowledge about preventable aspects of Type 2 diabetes (T2D), inaccurate information from lay sources, lack of trust in the healthcare system, and shortages of drugs due to financial constraints [45]. Atwine & Hjelm also reported skepticism from patients regarding a potential role for clinical health professionals in T2D management [46]. For this reason, Azevedo believes that an uncompromised healthcare system that identifies ways to trim financial and human resource waste and revamps the education system with targeted and measurable health outcomes is vital [47].

Low engagement with diabetes self-management programs limits their ability to reduce diabetes costs and provide effective and efficient control of diabetes. Thus, targeted implementation strategies can increase community engagement by identifying factors that increase uptake and adherence. Motivators for intervention participation may also influence retention. Our study participants were all interested in improving their health. Simacek et al. found that participants want to directly apply research findings to their everyday lives [48]. Additionally, we found that incentives such as transportation allowances and glucose monitoring kits could motivate patients. Despite care providers frequently perceiving diabetes patients as having a fatalistic attitude toward their health and not taking responsibility for their health, studies have found that financial incentives can improve retention in diabetes care [48,49]. However, some participants stressed that this should not be the reason for participation in the MSD program. Recent qualitative research revealed that patients might be motivated differently based on their circumstances and commitment to their treatment [49].

Another significant finding of the study was the participants’ desire for support from family members and peers with diabetes. People with diabetes are generally responsible for managing their blood glucose levels and diabetes treatment. However, peer and family support can play a pivotal role in convincing patients to modify their lifestyle, reduce the stress caused by a chronic disease, and improve compliance with medication. A randomized control trial comparing family-directed versus individual diabetes interventions found improved blood glucose levels, insulin sensitivity, and lipid profiles among the family-directed group. In addition, the metabolic risk increased for individuals but was maintained for participants who participated with family members [50]. Finally, there is evidence that mobile phone applications such as WhatsApp, as recommended by participants in this study, can remind patients of their medications and support their adherence. The use of simple and accessible technologies has shown high acceptance in many SSA settings [51,52]. Facilitators can communicate directly with intervention participants using social media approaches, overcoming the limitations of relying solely on in-person interactions.

**Strengths and limitations of the study**

Using HCD principles and approaches, we designed formative research that employed rigorous methods to understand participant needs to inform the development of a culturally appropriate diabetes control intervention for residents of Cotonou, southern Benin. Moreover, we found that many of the barriers and facilitators in this study were comparable to previous research studies, indicating that these themes are consistent across demographic groups. However, given the formative design and a limited number of FGDs, specific recommendations from this study may not be generalizable across contexts. Despite this limitation, we achieved some degree of saturation in the results during the analysis of the FGD data. Another limitation is the lack of rural comparison groups because participants from this study were mainly residents of a large urban city. Lastly, if focus group participants felt they could not express personal barriers, social desirability bias may have occurred.

**Implications for developing the community-based MSD in the Benin context**

The following insight statements highlight participants’ preferences for a diabetes management curriculum or program. These insights will be further used to shape and adapt the proposed MSD-Benin curriculum and program.

- Format: Program participants value group learning format as a supportive measure and shared experiences even within a self-directed diabetes management curriculum. Groups can integrate naturally occurring societal influences into the program and provide channels for developing key processes within communities [53]. However, this kind of family- and community-oriented approach will also inevitably require making at least some of the group sessions available to other family members to attend and be involved. Additionally, peer-to-peer influences could be developed naturally to provide encouragement and assistance in reaching those who otherwise might not avail themselves of the program [53]. These factors provide a strong rationale for a peer support model, as has been recently proposed [53].
- Strategy: Program participants value a mixture of materials, such as audiovisuals, mobile phone applications, and written materials, to deliver interventions. They preferred materials to be pictorials and in local languages within the intervention context. As such, cultural adaptations of Benin foods with pictorial preparation guides and physical activities will be acceptable in the country. It will be crucial to use participatory research methods to engage patients and community residents in adapting to the community health worker’s guidelines and tools for recommended activities so that they are linguistically and culturally appropriate, including guidelines for food consumption using locally available foods. These adaptations will need to use more graphics and photographs to be suitable for low-literacy participants.
- Delivery: Participants perceived community health workers (CHWs) to be the most effective in delivering the intervention, but they needed specialists like dieticians and clinicians who could co-facilitate certain aspects of the curriculum, suggesting a team leadership approach to the program delivery. For example, a trained diabetic educator (DE) can lead the didactic portions of group sessions, concentrating on the presentation of clinical and management aspects of diabetes and hypertension. Like CHWs already supporting families in improving maternal and child health outcomes in Benin, they will have responsibility for leading the group activities; role plays, demonstrations, and any community activities, as well as home visits and one-on-one visits support of participants. The training materials will need to be prepared in French, but the CHWs and DEs will be trained in presenting them in French and the local languages.

## 5. Conclusions

In Benin, long-term commitment to diabetes care and lifestyle changes is challenging. Hence, interventions that include sustained motivation specifically targeted at this population are urgently needed. The information and examples offered in this paper and the proposed MSD-Benin curriculum are essential for developing culturally appropriate interventions. This study provides a basis for building preventative and therapeutic approaches to diabetes management grounded in the needs and perspectives of program stakeholders, including patients. Additionally, it contributes to the literature on rigorous adaptation processes for introducing and implementing evidence-based interventions in new settings based on Human-centered design principles.

## Data Availability

The datasets used and/or analyzed during the current study are available from the corresponding author upon reasonable request.
